# Sensorimotor restriction affects complex movement topography and reachable space in the rat motor cortex

**DOI:** 10.3389/fnsys.2014.00231

**Published:** 2014-12-12

**Authors:** Mirco Budri, Enrico Lodi, Gianfranco Franchi

**Affiliations:** Section of Human Physiology, Department of Biomedical and Specialty Surgical Sciences, University of FerraraFerrara, Italy

**Keywords:** long-duration ICMS, forelimb immobilization, complex movement, kinematics, reachable space

## Abstract

Long-duration intracortical microstimulation (ICMS) studies with 500 ms of current pulses suggest that the forelimb area of the motor cortex is organized into several spatially distinct functional zones that organize movements into complex sequences. Here we studied how sensorimotor restriction modifies the extent of functional zones, complex movements, and reachable space representation in the rat forelimb M1. Sensorimotor restriction was achieved by means of whole-forelimb casting of 30 days duration. Long-duration ICMS was carried out 12 h and 14 days after cast removal. Evoked movements were measured using a high-resolution 3D optical system. Long-term cast caused: (i) a reduction in the number of sites where complex forelimb movement could be evoked; (ii) a shrinkage of functional zones but no change in their center of gravity; (iii) a reduction in movement with proximal/distal coactivation; (iv) a reduction in maximal velocity, trajectory and vector length of movement, but no changes in latency or duration; (v) a large restriction of reachable space. Fourteen days of forelimb freedom after casting caused: (i) a recovery of the number of sites where complex forelimb movement could be evoked; (ii) a recovery of functional zone extent and movement with proximal/distal coactivation; (iii) an increase in movement kinematics, but only partial restoration of control rat values; (iv) a slight increase in reachability parameters, but these remained far below baseline values. We pose the hypothesis that specific aspects of complex movement may be stored within parallel motor cortex re-entrant systems.

## Introduction

Several studies have provided compelling evidence that long-duration intracortical microstimulation (ICMS) with 500 ms of current pulses delivered to the motor cortex (M1) enables consistent mapping of complex movement categories across animals (monkeys: Graziano et al., 2002a,b, 2005; Gharbawie et al., 2011; rodents: Haiss and Schwarz, 2005; Ramanathan et al., 2006; Harrison et al., 2012; Bonazzi et al., 2013). Collectively, these studies suggest that the M1 is organized into several spatially distinct functional zones, and that these zones intrinsically organize movements that bear resemblance to the behavioral repertoire of the species (Graziano et al., 2002a,b). This view of M1 organization raises several unanswered important questions (Kaas et al., 2013). In particular, the issues of whether sensorimotor experience modifies the extent of representation of complex coordinated movements, or rather the intrinsic kinematics of ICMS-evoked movement, and the movement end-point into extrinsic space.

Previous studies, carried out with short-duration ICMS (with 30 ms of current pulses), provide evidence that M1 features change in relation to the amount of specific behavioral use of a body part (Kleim et al., 1998; Sanes and Donoghue, 2000). Namely, over/under-trained limb parts are over/under-represented within a preserved forelimb map size (Kleim et al., 1998; Milliken et al., 2013). Recent research in rats also suggest that after weeks of whole-limb disuse, M1 excitability profoundly deteriorates, so that the corresponding area of the limb map shrinks without being filled by neighboring cortical representation, leaving parts of the limb representation unresponsive to electrical stimulation (Langlet et al., 2012; Viaro et al., 2014). Moreover, weeks of limb disuse appears to result in altered synaptic connectivity (Viaro et al., 2014) and dendritic spine remodeling in the corresponding M1 regions (Trinel et al., 2013), indicating relevant changes in sensorimotor cortical processing. However, the short-duration ICMS applied in these studies is unable to characterize either the organization of functional zones within the M1, or the complexity of cortical motor control of voluntary movement (Schieber, 2001).

Previous studies in human patients have found that immobilization impairs complex aspects of voluntary movement, including altered trajectories, accelerations and inter-joint coordination (Moisello et al., 2008; Lissek et al., 2009). These impairments affecting feed-forward regulation of movement have been correlated with changes in limb internal models in the sensorimotor cortex (Kaneko et al., 2003; Huber et al., 2006; Moisello et al., 2008; Lissek et al., 2009; Roll et al., 2012). Whether such kinematic alterations of cortical motor control could be directly studied in animals and correlated to complex movements representation has not been thoroughly explored.

Short-duration ICMS studies (Langlet et al., 2012; Viaro et al., 2014) raise the question of how sensorimotor restriction impairs the representation of complex movement in M1, as evidenced by long-duration ICMS in rats (Bonazzi et al., 2013). To this end, we performed long-duration ICMS to define and quantify the changes in the topography and kinematics of complex movements, as well as changes in the map of target locations for the paw in extrinsic space, after weeks of forelimb immobilization.

## Materials and methods

### Ethical approval

Adult male Wistar rats (*n* = 20; 13–15 weeks old at cast application; 300–350 g; S.Pietro al Natisone, Harlan, Italy) were kept under regular lighting conditions (12 h light/dark cycle) and given food and water *ad libitum*. This study was compliant with the European Council Directive of 24th November 1986 (86/609/EEC), and approved by the University of Ferrara Ethics Committee and the Italian Ministry of Health. Adequate measures were taken to minimize both animal pain and the number of animals used.

### Experimental design

To analyze M1 changes that appear after unilateral forelimb immobilization, cortical output was evaluated by long-duration ICMS at different time points, i.e., after 30 days of immobilization, (Cast group, *n* = 7) and 14 days after cast removal (After-cast group, *n* = 6). In the former case, ICMS was performed at least 12 h after cast removal to ensure good forelimb reperfusion and to exclude the acute effects of reperfusion edema. Non-immobilized rats were used as controls (Control group, *n* = 7).

### Limb immobilization

Unilateral immobilization of the forelimb and forepaw was induced in ketamine-anesthetized rats, similarly to that described previously (Viaro et al., 2014). Briefly, the selected forelimb (alternating left and right) was first wrapped in a thin layer of cotton, to prevent compression, and subsequently blocked with a plaster bandage (Platrix; BSN medical, Milan, Italy). During this procedure, the digits were maintained in full extension in order to prevent obstruction of normal blood flow. The limb was plastered against the chest in a natural joint position, and the bandage was used to form a one-holed vest around the upper trunk. The limited movement of the confined limb imposed increased use of the contralateral (unconstrained) forelimb to improve posture, gait, and grooming. During the immobilization period, all rats were brushed and swabbed with a wet pad twice a day.

Although it was beyond the scope of this investigation to discover whether induced forelimb immobility influences stress and behavior, chronic immobilization is known to induce stress in rodents (Ghosh et al., 2013). Indeed, as a stress indicator, we observed an increase in the use of the ipsilateral-to-cast hindlimb to scratch the portion of cast closest to the neck in the immobilized animals. However, neither overall exploratory behavior nor food intake seemed to be affected at any time during immobilization. The ongoing use of the ipsilateral-to-cast hindlimb to scratch the neck and the hemi-face continued for 24–48 h after cast removal.

### Long-duration intracortical microstimulation

**Figure 1:**
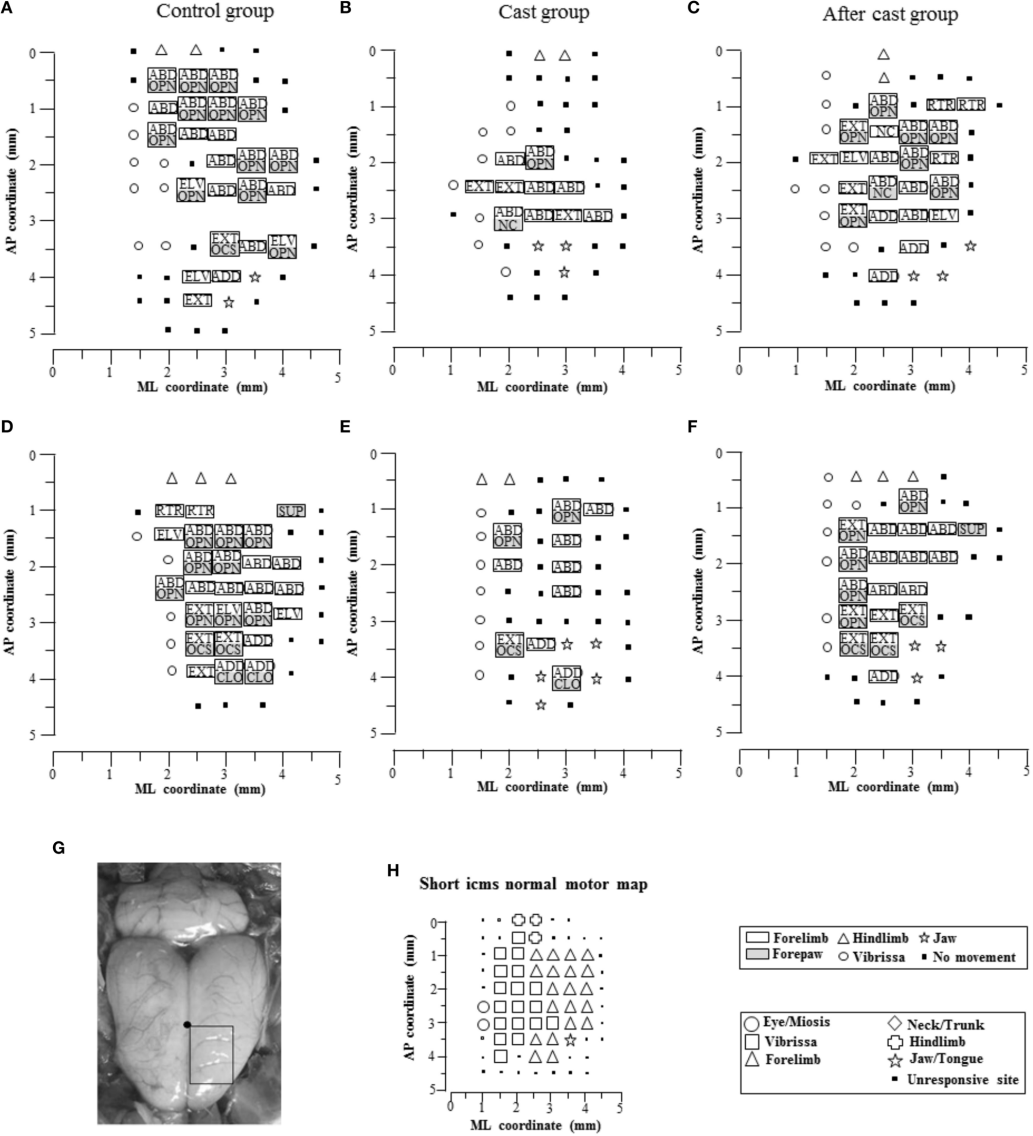
**Representative experiments in which the arrangement of sites across the cortical surface evoked complex forelimb movements in Control (A,D, rat 1 and 2 in **Table 2**), Cast (B,E, rat 5 and 6 in **Table 2**) and After cast (C,F, rat 3 and 6 in **Table 2**) groups**. The microelectrode was sequentially introduced to a depth of 1500 μm, and movements were evoked with stimulation intensity of 100 μA. Interpenetration distances were 500 μm. In these mapping schematic diagrams, frontal poles are at the bottom. Zero corresponds to the bregma, and numbers indicate rostral distance from the bregma or lateral distance from the midline. The type (limb or paw) of forelimb movement is indicated by the gray scale, and the class of forelimb movement by letters (see Table 1). Classes of limb movement: ABD, abduction; ADD, adduction; EXT, extension; RTR, retraction; ELV, elevation. Classes of paw movement: OPN, opening; CLO, closure; OCS, opening/closure sequence; SUP, supination; NC, not categorized. Sites that evoked movements of other body parts are indicated by different symbols; non-responsive sites are depicted by small filed squares. Non-forelimb movements simultaneous with forelimb movements are not represented. The absence of a symbol (within or at the border of the maps) indicates that no penetration was performed due to the presence of a large blood vessel. Note that the size of symbols used to identify the movement in each site does not define the size of the representation of movements. The lower left photograph **(G)** shows a top view of the rat cortical surface. The rectangle superimposed illustrated the approximate location of the area under study relative to bregma (filled small circle on the midline). The schematic in the center of the lower row **(H)** shows a representative experiment in which normal topography of motor cortex was mapped with standard ICMS with short-duration pulses.

For mapping procedures, rats were initially anesthetized with ketamine HCl (80 mg/Kg, i.p.). For the duration of the experiment, anesthesia was maintained through supplementary ketamine injections (4 mg/Kg, i.m., given as required, typically every 25–30 min) so as to achieve long-latency and sluggish hindlimb withdrawal after hindfoot pinching. Under anesthesia, the body temperature was maintained at 36–38°C using a heat lamp. ICMS mapping was performed to define the topographic distribution of complex forelimb movements in M1. The mapping procedure was similar to that described in a previous study (Bonazzi et al., 2013). In brief, animals were placed in a Kopf stereotaxic apparatus. The body of the animal was laid in a prone position on a desk with its forelimbs hanging down and free to move in all directions against gravity. The position of the trunk was stabilized to the back of the desk to minimize spontaneous trunk movements (head/chest-fixed coordinates). The resting position for each forelimb was in approximately halfway extension-adduction, and the wrist rested palm down with the finger joints in semi-extension. A large craniotomy was performed to expose the frontal cortex of one hemisphere. The dura remained intact and was kept moist with saline solution. Electrode penetrations were regularly spaced out over a 500 μm grid. It was sometimes necessary to alter the coordinate grid, by up to 70 μm, to prevent the electrode from penetrating a surface blood vessel. Glass-insulated tungsten electrodes (0.6–1 MΩ, impedance at 1 KHz) were used for stimulation. The electrode was lowered vertically to 1.5 mm below the cortical surface, corresponding to layer V of the frontal agranular cortex (Franchi, 2000). To evoke complex movements, at each cortical site studied, stimulation was applied by an S88 stimulator and two PSIU6 stimulus-isolation units (Grass, Quincy, Mass., USA). A 500-ms train of 200-μs-duration bipolar pulses was delivered at 333 Hz. Each stimulation pulse was obtained using biphasic current, in which a negative phase was followed by a positive phase, to minimize damage that could occur during long-duration stimulation (Graziano et al., 2002a). Current was measured as the voltage drop across a 1-KOhm resistor in series with return of the stimulus isolation units. The stimulating current was injected at each cortical site, starting from 20 μA and increasing gradually in 10 μA steps until a clear multi-joint movement of the forelimb was detected. Once multi-joint movement was detected (~50 μA), the current was raised to 100 μA to optimize movement and facilitate characterization, and then quantitative testing was begun. Movements were mapped so that all forelimb motor region was explored in all cases included in the study (see examples in Figure 1). If no movement was detected at 100 μA, the site was defined as non-responsive (Figure 1).

### Video recording of complex movements

Movements evoked by long-duration ICMS were visually identified during mapping sessions, and videotaped at 30 frames/s using a standard digital videocamera. This recording device was positioned so as to obtain a lateral or frontal view of the animal. In order to detect stimulus onset, a triggered LED was positioned near the body of the animal, within the visual field of the camera. The video recording served as a back-up to clarify the data analysis and provide information on the occurrence of forelimb movements. Videotaped movements were analyzed frame-by-frame using Quicktime and iMovie software (Apple Inc., Cupertino, USA). Video recording data showed good agreement with those obtained in the previous study (Bonazzi et al., 2013), and were therefore not represented in the Figures of this paper.

### Kinematic recording and analysis of complex movements

Movements evoked by long-duration ICMS were recorded and measured with a motion 3D-optical analyzer (Qualisys Motion Capture System; Qualisys North America Inc, Charlotte, USA). Two adhesive infrared-reflective spheres (diameter: 0.30 cm, weight: 0.04 g) were placed as markers at two anatomical landmarks on the forelimb skin, namely: (i) the dorsal middle of the wrist and (ii) the last phalangeal joint (tip) of the two middle digits, used, respectively, to detect the limb and paw movement. In all experiments markers were placed by the same operator in order to minimize positioning variability. Three infrared cameras, placed around the animals, were used to record the position of markers. The cameras were calibrated according to the Qualisys Motion Capture Analysis System procedure, placing a stationary L-shaped reference grid with 4 markers below the animals to define the origin and orientation of the 3D-coordinate system. The directions of X-, Y-, and Z-axes coordinates were anterior, lateral, and dorsal, respectively. Movements were recorded for 2 s at a sampling rate of 100 Hz, and kinematic features were analyzed off-line using Qualisys Track Manager software and custom MATLAB programs (The Mathworks Inc, Natick, USA). The forelimb starting position was not modified during any of the stimulation trials performed in the absence of spontaneous movement. In each animal, sham stimulations were performed, both while the animal was standing quietly with the forelimb stationary, and while moving it spontaneously. In order to avoid interference between spontaneous and evoked movements, ≥4 mm displacement of the wrist or digit marker in at least one of the X, Y, or Z axes was considered for analytical purposes. All measurements were made by subtracting the marker's resting position in Cartesian coordinates from all points along the trajectory, and thus all data sets began at (0,0,0). The initial analysis sought to define the classes of movement and their topography across the cortical surface (classes of movement: see Table 1 and movement evoked in each stimulated site: see Figure 1). Since a vertical component (Z-axis displacement) was found in all evoked limb movements, the maximal displacement in one of the X or Y axes defined the class of limb movement when the distance exceeded 15% of the displacement in the other axis. The Z-axis displacement defined the type of movement when X- and Y-axis displacement was <4 mm (i.e., movement cut-off). We found that these values were the most reliable ones for defining the type of forelimb movement in all animals. When either the X-, Y- or Z-axis displacement failed to meet the criteria defined above, the movement was defined as not classifiable (NC, see Results). When a paw movement was observed, each paw component was calculated by subtracting the wrist marker position value from the digit marker position value at all points throughout the movement. For each stimulated site, the kinematic variables were obtained by averaging the values obtained in 3–5 microstimulation trials. Analysis provides information on different and complementary movement patterns. Movement onset was defined as the time-point (in ms) at which the tangential velocity exceeded 5% of the maximum velocity (Adamovich et al., 2001), and the time-range between stimulation and onset of movement was taken as the movement latency (L in ms). The end of movement was defined as the time-point (in ms) at which the marker reached maximal displacement (in mm) in one of the X, Y, or Z axes. Displacement toward the resting position and outlasting the stimulus was not considered. The total time spent during movement was defined as the movement duration (D in ms). For each movement, we determined the mean velocity (MV in mm/s), and the maximal peak velocity (MPV in mm/s). A peak velocity was counted when it exceeded the mean velocity. For the wrist marker we also determined the trajectory (T in mm) and vector (DV in mm) from the initial to final position for each stimulated site. Limb trajectory straightness was determined by the path index (defined as trajectory/vector length ratio), assuming that a trajectory following a straight line has an index of 1, while a semicircular trajectory has an index of 1.57 (Archambault et al., 1999). In any case, a path index >1.57 was taken to indicate a trajectory whose curvature exceeds that of the arc of a circle.

**Table 1:** **Abbreviation and description of the elicited movements**.

**Abbreviation**	**Movement**	**Movement description**
**PROXIMAL**		
ABD	Abduction	The limb was raised and brought outwards from the midline (maximal displacement on Y-axis positive)
ADD	Adduction	The limb was raised and brought toward the midline (maximal displacement on Y-axis negative)
EXT	Extension	The limb was raised and brought forward (maximal displacement on X-axis positive)
RTR	Retraction	The limb was raised and brought backward (maximal displacement on X-axis negative)
ELV	Elevation	The limb was raised without shifting in another direction (maximal displacement on Z-axis positive; X and Y axes < 4 mm)
**DISTAL**		
OPN	Opening	Positive X-axis value
CLO	Closure	Negative X-axis value
OCS	Opening/closure sequence	Positive X-axis followed by negative X-axis values
SUP	Supination	Positive Z-axis value

### Data presentation and statistical analysis

**Figure 2:**
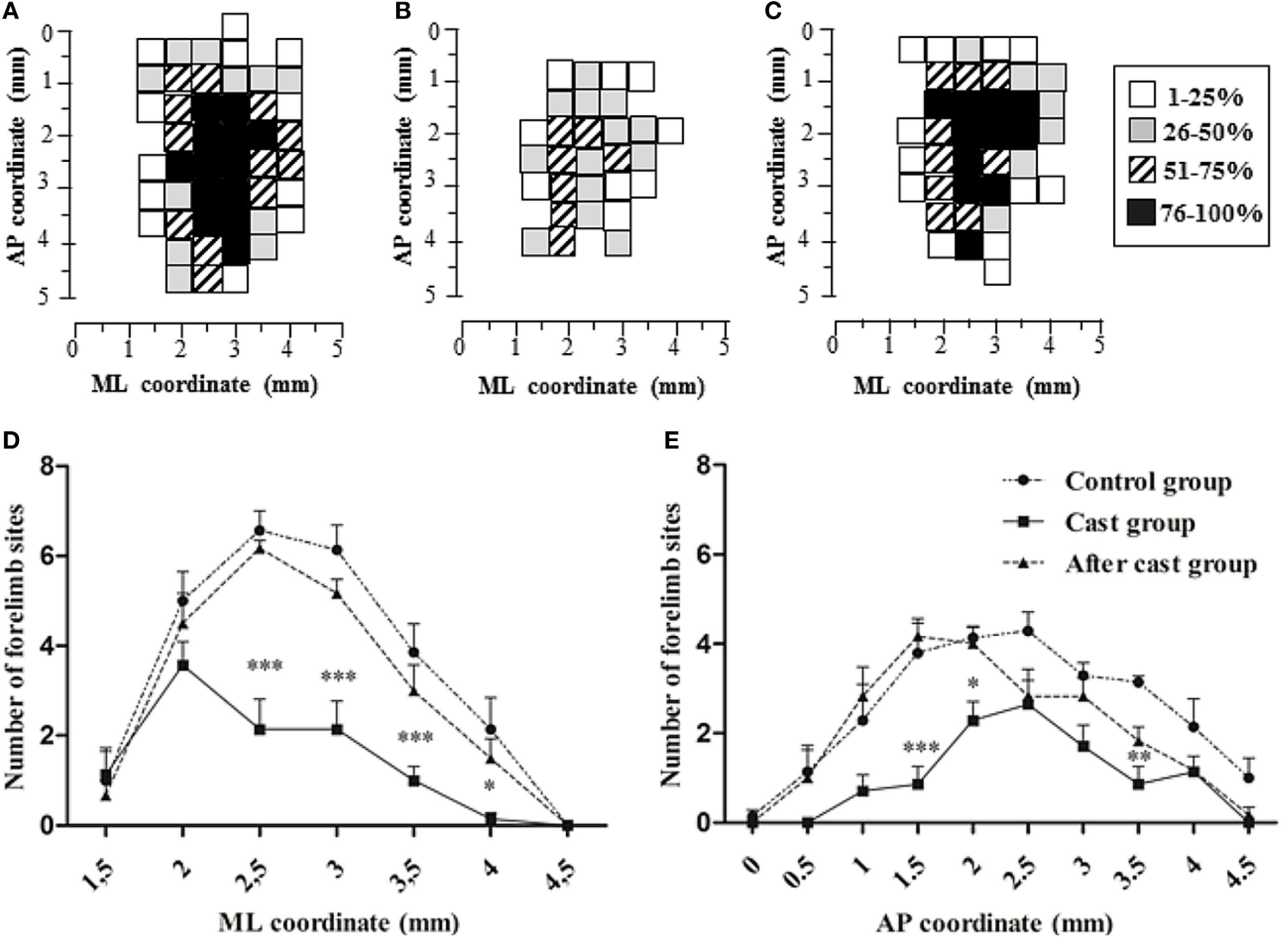
**Unilateral forelimb casting affected the representation of forelimb sites across the cortical surface**. Cumulative description of forelimb movements evoked in all animals in Control **(A)**, Cast **(B)**, and After cast **(C)** groups. The surface plot shows the frequency distribution of sites at each coordinate relative to the bregma. The frequency of movement at each site is coded on a gray scale, and 100% probability is achieved when a movement at that site was observed in all animals in a group. Medio-lateral **(D)** and antero-posterior **(E)** distribution of sites eliciting forelimb movement. Comparison between the distribution in Control vs. Cast and After cast groups. ^*^P < 0.05, ^**^P < 0.01, ^***^P < 0.001. Note that there was no difference in distribution between the After cast and Control groups (Two-Way ANOVA followed by contrast analysis and the sequentially rejective Bonferroni test for multiple comparisons).

**Figure 3:**
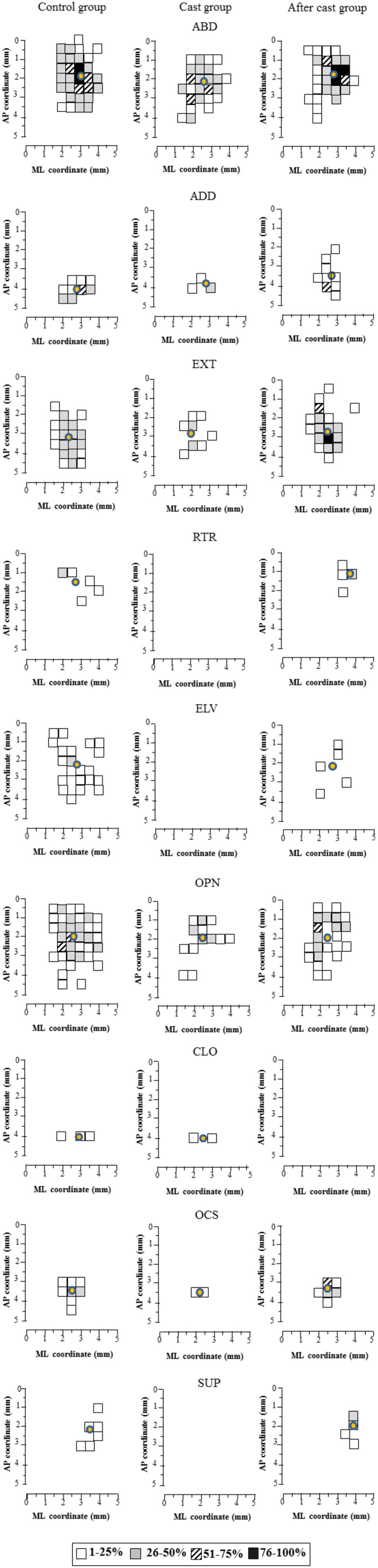
**Unilateral forelimb casting affected the representation of limb and paw classes of movement across the cortical surface**. The surface plot shows the frequency distribution of sites at each coordinate relative to the bregma. The rat group is indicated: Control group (left column maps), Cast group (middle column maps); After cast group (right column maps). Movement classes are indicated by abbreviations: ABD, abduction; ADD, Adduction; EXT, extension; RTR, retraction; ELV, elevation; OPN, opening; CLO, closure; OCS, opening/closure sequence; SUP, supination. See Figure 2 for cumulative map construction. Circle overlapping the map: CoG. Note that after long-term cast (middle column) the area pertaining to EXT and OPN significantly diminished in size; those representing RTR, ELV, and SUP disappeared. After cast, there was a recovery in limb and paw movement classes approaching that of controls except for CLO size. In addition, the selective reorganization of each functional zone did not involve a shift in the CoG.

All data are presented as mean ± s.e.m. of *n* determinations. To characterize the spatial distribution of all movements in the motor cortex surface across animals, a 2D distribution of movement-responsive sites at coordinates relative to the bregma was generated. Each movement-related site was taken to represent a 0.25-mm^2^ square of cortical surface (0.50 × 0.50 mm), and 100% probability at a given site was achieved when a movement was observed at that site in all animals (Figures 2A–C, 3). The center of gravity of the cortical representation was calculated for each class of movement (Figure 3, CoG; Littmann et al., 2013). For each group of rats, a quantitative two-dimensional evaluation of the forelimb motor cortex configuration was obtained by comparing medio-lateral and antero-posterior distribution of sites eliciting forelimb movements (Figures 2D,E). To this end, penetrations in each hemisphere were divided into 0.5-mm-wide bins into which all sites eliciting forelimb movement were grouped, irrespective of their medio-lateral or antero-posterior coordinates. To analyze between-group differences in forelimb site distribution, we applied Two-Way ANOVA followed by contrast analysis and the sequentially rejective Bonferroni test for multiple comparisons to each bin. A multivariate test for difference in means (MANOVA) was used to compare displacement on the X, Y and Z axes, as well as kinematic variables. Multivariate discriminant analysis (MDA) was used to analyze the X-, Y-, and Z-axis displacement and kinematic variables in order to classify movements into predefined classes. MDA is a useful tool to classify observations into more groups when a sample has a priori known groups (Hair et al., 1998). We classified the movements off-line starting from the displacement values in X, Y, and Z axes obtained from the Qualisys system and applying the criteria described above and summarized in the Table 1. We applied the multiple discrimination analysis to investigate how the maximal displacement values in X, Y, Z axis contributed to the group separation. With MDA, all independent variables are assumed to be normal and all groups are assumed to have the same covariance matrix. The algorithm classified each movement assigning every group a Z score obtained by weighting every predictor value (x,y,z in this analysis) for a coefficient in order to maximize the inter-group variance while minimizing the intra-group variance. A discriminant Z score for each observation was calculated by the following formula:

(1) $${\text{Z}}_{\text{jk}}={\text{a+W}}_{1}{\text{X}}_{\text{1k}}+{\text{W}}_{2}{\text{X}}_{\text{2K}}+\ldots +{\text{W}}_{\text{n}}{\text{X}}_{\text{nk}}$$

where

(2) $$\begin{array}{l} {\text{ Z}}_{\text{jk}}=\text{discriminant Z score of discriminant function j for}\\ \text{ object k}\\ \text{ a}=\text{intercept}\\ \text{ Wi}=\text{discriminant coefficient for independent variable i}\\ {\text{ X}}_{\text{ik}}=\text{independent variable I for object k} \end{array}$$

Then, in order to construct the classification matrices, it computed the optimum cutting score (a critical Z score) for each movement class. The optimal cutting score was the one that misclassified the fewest number of movement across all groups. For unequal groups a weighted average of the groups centroids provided an optimal cutting score for the discriminant function. The Minitab 15 program (Minitab, State College, USA) provided the discriminant score as well as the Z score for each group of movement.

Then the Minitab program compared the individual discriminant scores for each group with the critical cutting score value and classified as follows:

(3) $$\text{Classify an individual movement into group A if }{\text{Z}}_{\text{n}}<{\text{Z}}_{\text{ct}}$$

or

(4) $$\begin{array}{l} \text{Classify and individual movement into group B if }{\text{Z}}_{\text{n}}>{\text{Z}}_{\text{ct}}\\ {\text{Z}}_{\text{n}}=\text{discriminant Z score for the nth individual movement}\\ {\text{Z}}_{\text{ct}}=\text{critical cutting score value} \end{array}$$

The Minitab 15 program presented the classification procedures in matrix form. The matrix shows for every group the number of individual movements correctly assigned to his group by the discriminant function. The measure of confidence for the corrected classification and the measure of the predicted error rate for each classification were defined by: Proportion correct = Number of correctly classified samples/Total number of samples; Error rate = Number of rejected samples/Total number of samples. To better characterize changes in the target movement locations in space, we defined the 3D distribution of limb movement endpoints in each group of animals (**Figure 7**). In a further analysis, we assumed that the 3D plot of the endpoints of all limb movements for each group could directly show the 3D representation of the ICMS-defined reachable space (**Figure 8**). In this way, by measuring the volume in which endpoints were enclosed and its surface area, we could show how reachable space could be changed in the Cast and After-cast group vs. the Control group. To this end, the ICMS-defined reachable space was defined as the portion of the 3D confidence ellipsoid in relation to the distribution

(5) $$\left[\begin{array}{c} P\\ NP \end{array}\right],\text{where }P\;=\;\left[\begin{array}{ccc} x_{1} & y_{1} & z_{1}\\ x_{2} & y_{2} & z_{2}\\ \cdots & \cdots & \cdots \\ x_{n} & y_{n} & z_{n} \end{array}\right]\text{and }NP\;=\left[\begin{array}{ccc} -x_{1} & -y_{1} & -z_{1}\\ -x_{2} & -y_{2} & -z_{2}\\ \cdots & \cdots & \cdots \\ -x_{n} & -y_{n} & -z_{n} \end{array}\right].$$

By applying principal component analysis (PCA) to the matrix (custom-made MATLAB programs) it was possible to calculate the 3D coordinates of an ellipsoid whose center was the center of the XYZ axes and whose volume represented 95% probability of containing movement endpoints. From the resulting probability ellipsoid, the reachable space was defined by that part of ellipsoid containing endpoints *P* with XYZ ≥ minimum values. To analyze differences in kinematic means, One-Way ANOVA followed by contrast analysis and the sequentially rejective Bonferroni test for multiple comparisons were performed when the number of sites was ≥ 7 in two out of three groups of animals. All statistical procedures were carried out using Minitab 15 and MATLAB (The Mathworks Inc., Natick, USA) software. Values of *p* < 0.05 were considered statistically significant.

## Results

### General observations

ICMS and ICMS-evoked forelimb movement recording were performed (see Table 2) in control rats (Control group *n* = 7), and in rats after 30 days of forelimb casting (Cast group, *n* = 7) and 15 days after cast removal (After cast group, *n* = 6). In all animals elicited movements proved to be repeatable between trials, and their features remained nearly constant over the time required to characterize each cortical site. The stimulation preferentially evoked forelimb movement contralateral to the stimulated hemisphere; although bilateral movements were occasionally evoked but ipsilateral movements were not. In order to enable consistent movement characterization between rat groups, all stimulations were performed at 100 μA. Stimulation in sites located on the periphery of the forelimb region generally evoked additional movements, which were, however, not studied systematically. These additional movements began at the same time as forelimb movements, and involved the hindlimb, whiskers, neck, eye, and/or jaw. The type of additional movement evoked depended on the forelimb-surrounding representation nearest to the stimulated point. For example, the additional movement evoked in sites located in the medial part of the forelimb representation involved the simultaneous bilateral activation of whisker and neck muscles. Stimulation of these sites frequently led to all contralateral whiskers being pulled backward and all ipsilateral whiskers forward. Only occasionally were neck twitches and whisker movements accompanied by saccadic-like movement toward the retracted whiskers. Together, this complex movement appeared as a coordinated orienting-like response toward the side contralateral to the cortex to be stimulated. In other sites located in the rostral-lateral part of the forelimb cortex, jaw/tongue movements accompanied forelimb movements, so that collectively this movement appeared as a coordinated bringing-to-mouth behavior.

**Table 2:**
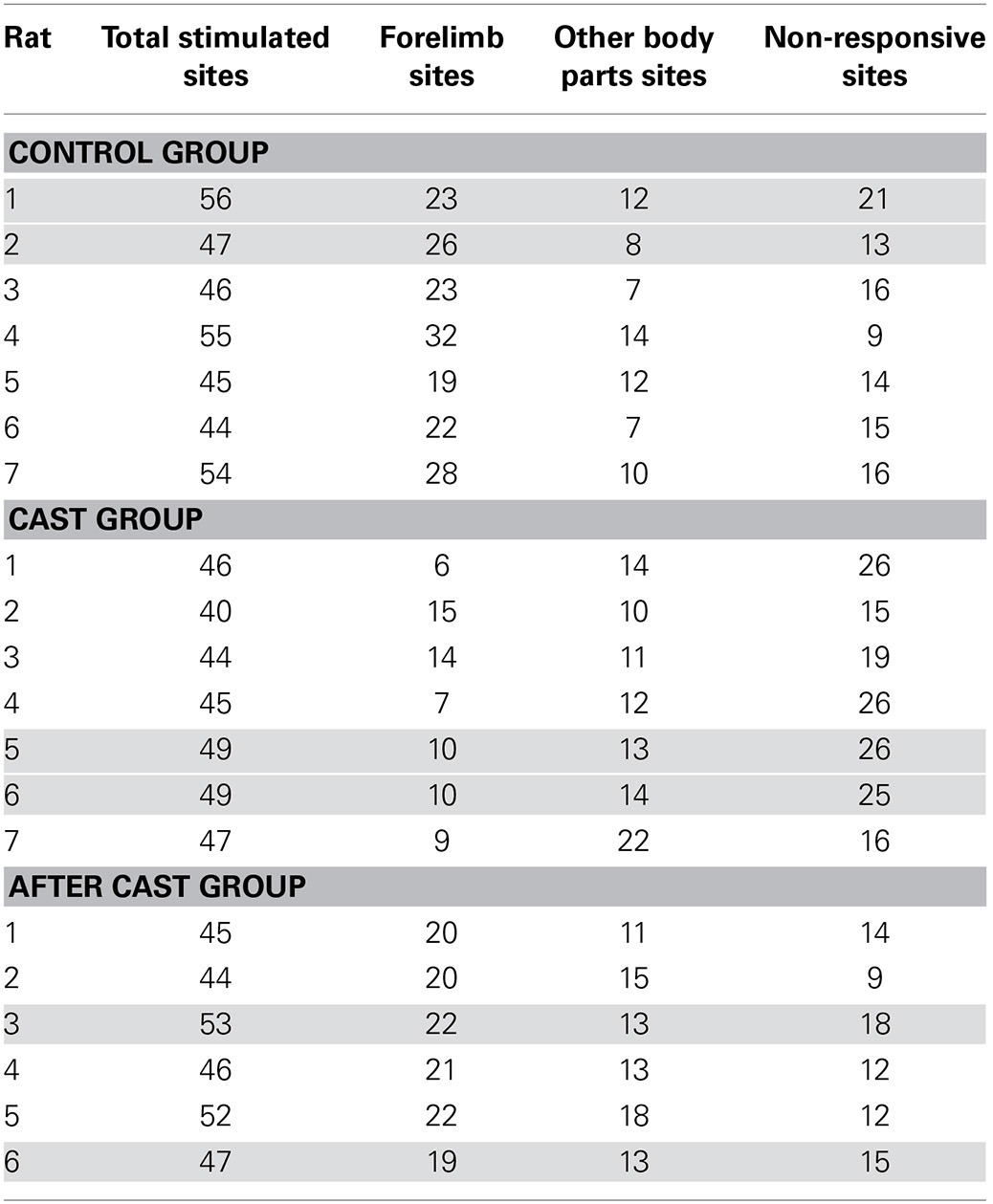
**Table showing individual cases in each experimental group**.

The number of sites are depicted in different columns. Rows in gray correspond to the cases shown in Figure 1.

### Normal organization of complex movement in forelimb motor cortex

Overall, forelimb movements were evoked at 173 sites, (mean in each animal: 24.71 ± 1.63; Table 2 and Figure 1). Forelimb movements were characterized by movement of one or both markers. The limb movement (wrist marker) was evoked in 92 of the sites (mean in each animal: 13.14 ± 1.10), paw movement (digit marker) in 6 (mean in each animal: 0.86 ± 0.26), and the limb-paw movement combination in the remaining 75 sites (mean in each animal: 10.71 ± 1.29). In control animals, the types of forelimb movement evoked were similar to those detailed in our previously published report (Bonazzi et al., 2013). The repertoire of limb movements evoked by prolonged stimulus trains included: (i) abduction (ABD): the limb was raised and brought outwards from the midline (maximal displacement on Y axis: positive; *n* = 83, 49.70%); (ii) adduction (ADD): the limb was raised and brought toward the midline (maximal displacement on Y axis: negative; *n* = 15, 8.98%); (iii) extension (EXT): the limb was raised and brought forward (maximal displacement on X axis: positive; *n* = 37, 22.16%); (iv) retraction (RTR): the limb was raised and brought backward (maximal displacement on X axis: negative; *n* = 6, 3.59%); (v) elevation (ELV): the limb was raised without shifting in another direction (maximal displacement on Z axis: positive and X and Y axes < 4 mm; *n* = 22, 13.17%); (vi) not categorized (NC): the limb displacement value on the X, Y, and Z axes did not match criteria defining the movement type (see Materials and Methods; *n* = 4, 2.40%). As in the previous study (Bonazzi et al., 2013), multivariate discriminant analysis demonstrated that we achieved a high rate of correct classifications (predictors: X-, Y-, and Z-axis values vs. movement class, 79.64%, 133 out of 167 movements; *p* < 0.0001), and also revealed that the 20.36% false alarm rate was largely due to some ABDs (23 out of 83), EXT (6 out of 37) and NCs (2 out of 4) that the classifier recognized as ELVs, NCs and ADDs, respectively.

The repertoire of paw movements included: (i) paw opening (OPN; positive X-axis value; *n* = 60, 74.07%); (ii) paw closure (CLO; negative X-axis value; *n* = 3, 3.70%); (iii) paw opening/closure sequence (OCS; positive X-axis followed by negative X-axis values; *n* = 10, 12.35%); (iv) paw supination (SUP; positive Z-axis value; *n* = 6, 7.41%), and (v) not categorized (NC): the paw displacement value on the X, Y, and Z axes did not match the criteria defining the movement type (see Materials and Methods; *n* = 2, 2.47%). OPN, CLO, and OCS were characterized by simultaneous contraction of the digits, while SUP showed external rotation of the wrist with no digit movement. Multivariate discriminant analysis demonstrated the achievement of a high correct classification rate (predictors: X-, Y-, and Z-axis values vs. movement class, 71.43%, 65 out of 91 movements; *p* < 0.0001), and showed that false alarms (28.57% of movements) were largely due to low discrimination between OPN and the OCS opening phase (classification rate: 65.00 and 60.00%, respectively). Multivariate discriminant analysis demonstrated a 100% correct classification rate for CLO and SUP. Given the low number of sites, the NC limb and paw movements had not been considered in the statistical analysis of a previous work (Bonazzi et al., 2013), but was added in this case to enable comparison of the control group with the Cast and After-cast groups. In controls, kinematic features of movement were similar to those demonstrated in previous study (Bonazzi et al., 2013), and were used for statistical comparison with the experimental groups of animals (see below). In control animals, representational maps of complex forelimb movements were consistent from one animal to the next, as previously demonstrated in previous study (Bonazzi et al., 2013). The map data of control cases were used for statistical comparison with that of animals subjected to movement restriction (see below).

### Long-term cast altered complex movement representation

The long-term forelimb cast caused a substantial reduction in the number of sites at which complex forelimb movement was evoked, as compared to control (total sites *n* = 71, mean in each animal: *n* = 10.14 ± 1.26, *p* < 0.001; Table 2 and Figure 1). It also reduced the number of sites at which limb (*n* = 46, mean in each animal: *n* = 6.57 ± 0.95, *p* < 0.001) and limb-paw movement (*n* = 25, mean in each animal: *n* = 3.57 ± 0.65, *p* < 0.001) were evoked, together with the disappearance of sites from which paw movement was evoked as an individual movement (Figure 1). To evidence the effect of the cast on the spatial distribution of forelimb sites across the cortical surface, a bregma-relative 2D frequency distribution of movement sites was generated, in which cumulative sites were coded according to their rates (Figures 2A–C). After long-term casting the cumulative map lacked higher frequency sites (76–100%, Figure 2B vs. Figure 2A). This effect was particularly marked when the number of forelimb sites in each bin of the map were plotted according to their medio-lateral (Figure 2D) or antero-posterior coordinates (Figure 2E). These distributions showed that responsive sites were reduced across the entire cortical representation, except at sites located medially (between 1.5 and 2 mm) in the ML distribution (Figure 2D).

Discriminant analysis showed that evoked movements in cast-immobilized rats were well classified using the control criteria. The repertoire of limb movements included: (i) ABD, *n* = 50, 70.42%; (ii) ADD, *n* = 4, 5.63%; (iii) EXT, *n* = 11, 15.49%; and (iv) NC, *n* = 6, 8.45%, while there was no evidence of ELV or RTR movements. As in controls, multivariate discriminant analysis revealed a high correct classification rate (predictors: X-, Y-, and Z-axis values vs. movement class, 91.55%, 65 out of 71 movements; *p* < 0.0001), also showing that the 8.45% false alarm rate was largely due to some NCs (3 out of 6) that the classifier recognized as ADD (*n* = 2) or ABD (*n* = 1). The repertoire of paw movements included: (i) OPN, *n* = 18, 72.00%; (ii) CLO, *n* = 2, 8.00%; OCS, *n* = 2, 8.00%; and (iv) NC, *n* = 3, 12.00%, while there was no evidence of SUP movements. In this case too, multivariate discriminant analysis revealed a high correct classification rate (predictors: X-, Y-, and Z-axis values vs. movement class, 81.48%, 22 out of 27 movements; *p* < 0.0001). The false alarm rate (18.52% of movements) was due to some OPNs (5 out of 18) that the classifier recognized as the OCS opening phase.

To evidence the effect of cast-immobilization on the spatial distribution of each class of movement, a 2D frequency distribution of sites was generated, in which cumulative sites were coded according to their rates (Figure 3). These cumulative maps, generated for each class of limb and paw movement, show the direct relationship between changes in representations and different zones of the forelimb motor cortex. In order to evaluate a possible representational shift, the center of gravity for each class of movement was monitored. This additional analysis clearly showed that the shrinkage in the representation coincided with a slight displacement of the center of gravity (Figure 3), but that this was always below the mapping resolution of 500 μm (mean shift in antero-posterior coordinate: 149.17 ± 65.42 μm; mean shift in medio-lateral coordinate: 259.17 ± 33.80 μm). After long-term cast-immobilization, the area pertaining to EXT diminished in size, while those representing ABD and ADD showed a not significant decrease (Cast vs. Control mean size in mm^2^, ABD: 1.79 ± 0.37 vs. 2.96 ± 0.49, *p* > 0.05; ADD: 0.14 ± 0.09 vs. 0.54 ± 0.13, *p* > 0.05; EXT: 0.39 ± 0.09 vs. 1.32 ± 0.29, *p* < 0.05; Figure 1) and those representing RTR and ELV disappeared altogether (Control, RTR: 0.21 ± 0.09, ELV: 0.79 ± 0.18). Likewise, OPN area significantly shrank, while OCS and CLO showed a not significant decrease in size (Cast vs. Control mean size in mm^2^; OPN: 0.64 ± 0.17 vs. 2.14 ± 0.29, *p* < 0.01; OCS: 0.07 ± 0.05 vs. 0.36 ± 0.09, *p* > 0.05; CLO: 0.07 ± 0.05 vs. 0.11 ± 0.07, *p* > 0.05), and SUP lost its representation. In addition, NC movement showed a not significant increase in its cortical representation size (Cast vs. Control limb mean size in mm^2^, NC: 0.21 ± 0.11 vs. 0.14 ± 0.14, *p* > 0.05; paw mean size in mm^2^, NC: 0.11 ± 0.05 vs. 0.07 ± 0.07, *p* > 0.05). It should be mentioned that some comparisons failed to reach significance in these analyses due to large inter-animal variability.

### Long-term cast altered the representation of combined limb-paw movements

**Figure 4:**
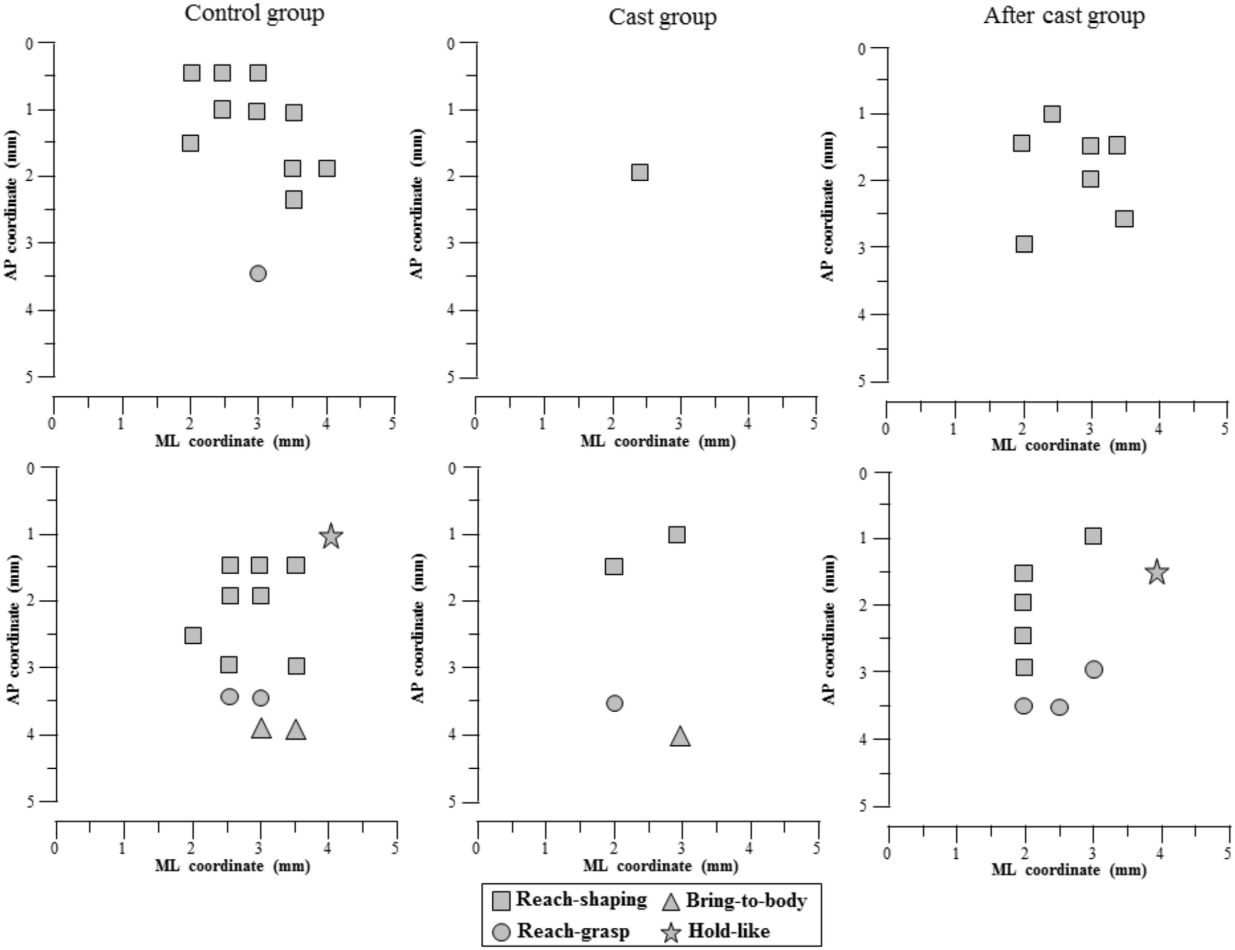
**Unilateral forelimb casting affected the representation of limb-paw movements that bear resemblance to the behavioral repertoire**. Representative arrangement of the four categories of limb-paw movement across the cortex in Control, Cast and After cast groups. Sites are symbol coded according to the category of movement evoked. Sites where stimulation failed to produce paw movement have been excluded. See Figure 1 for map construction. Note that in Cast group *reach-shaping* movement covered a smaller cortical region than in Control and After cast groups and *hold-like* movement was not found.

Prolonged train stimulation evoked movements involving in specific combination both forelimb and forepaw (Bonazzi et al., 2013 and current data). As in a previous study, we identified four categories of limb-paw movements in control animals (Figure 4), all of which tended to be evoked from different zones of the forelimb motor cortex, namely: *reach-shaping*: the limb was brought outward laterally or in front of the chest (ABD or EXT and ELV, respectively) coinciding with opening of the paw; *reach-grasp*: the limb was brought in front of the chest (EXT) coinciding with a repetitive sequence of paw opening/closing time-synchronized with the acceleration-deceleration phase of the reaching; *bring-to-body*: the limb was brought to the body (ADD) while the paw closed; and *hold-like* movement: the paw turned (SUP), orienting the palm toward the midline or face. The *reach-grasp* and *bring-to-body* movements were found in the rostral part of the forelimb motor cortex, whereas the *hold like* movement was located laterally to other movements (Figure 4). After long-term cast-immobilization (Figure 4), the motor cortex of all animals lost its representation of *hold-like* movement (Control:0.21 ± 0.07). The *reach-shaping* movement was detected, but its representation covered a smaller cortical region with respect to controls; the *reach-grasp* and *bring-to-body* movements were observed at two sites in two out of seven animals (Cast vs. Control mean size in mm^2^; reach-shaping: 0.57 ± 0.17 vs. 1.68 ± 0.23, *p* < 0.01; reach-grasp: 0.07 ± 0.05 vs. 0.36 ± 0.09, *p* > 0.05; bring-to-body: 0.07 ± 0.05 vs. 0.11 ± 0.07, *p* > 0.05).

### Long-term cast altered complex movement kinematics

**Figure 5:**
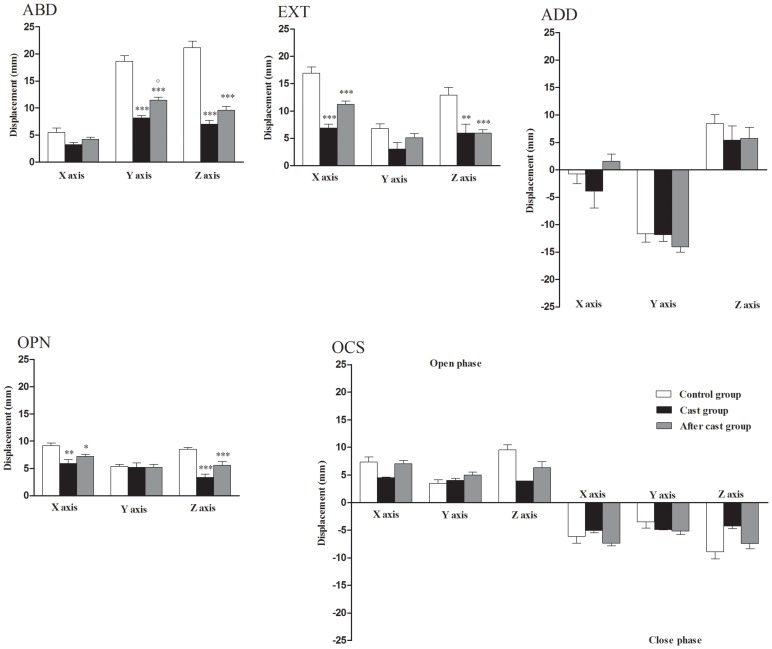
**Unilateral forelimb casting affected maximum displacement in X, Y, and Z axes for limb and paw classes of movement**. The maximum displacement values were considered for comparative statistical analysis only when a class was observed at least seven sites in two out of three groups of animals. ABD: abduction, EXT: extension, ADD: adduction, OPN: opening, OCS: opening/closing phase. Note that in ABD, EXT, and OPN, the effect significantly involved the axis labeling the class of movement, OCS showed a trend, whereas this effect did not emerge in ADD. Statistical comparison: ^*^P < 0.05, ^**^P < 0.01, ^***^P < 0.001, Cast and After cast different from Control group; °*P* < 0.05, After cast different from Cast group (ANOVA followed by contrast analysis and the sequentially rejective Bonferroni test for multiple comparisons).

**Figure 6:**
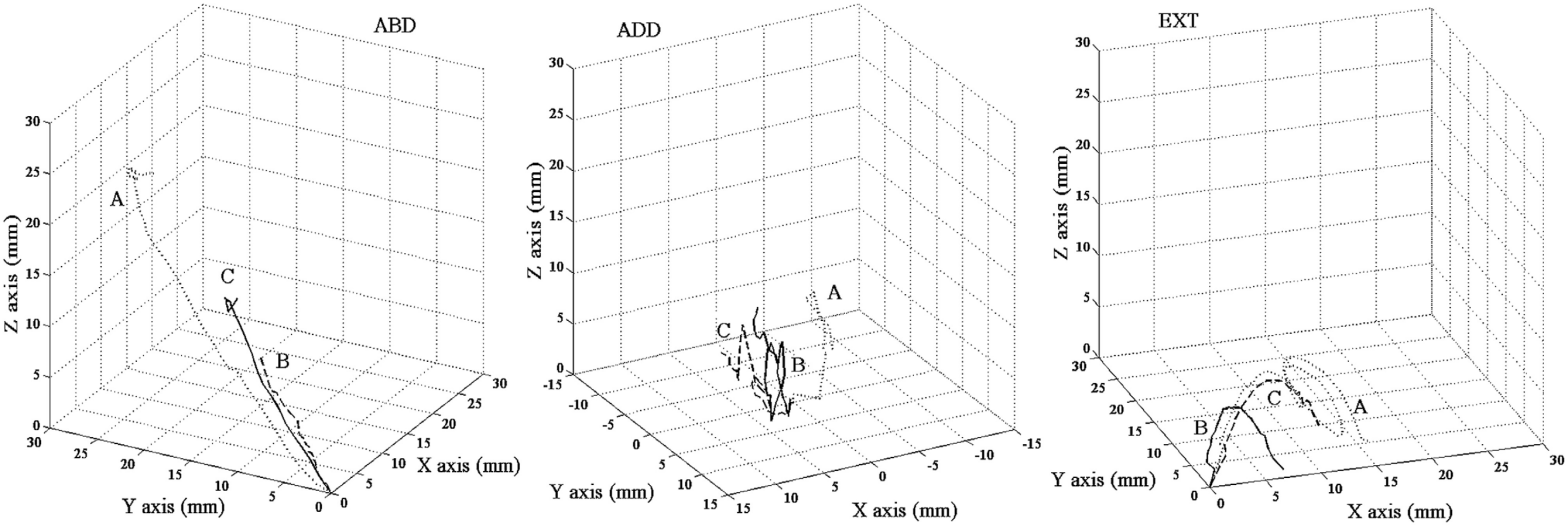
**Unilateral forelimb casting affected the trajectory length and shape**. Three-dimensional representation of trajectories with path index <1.57 (ABD) and >1.57 (ADD and EXT). The trajectories correspond to examples of individual movement evoked by a single ICMS pulse. The rat group is indicated by a capital letter: A, Control group; B, Cast group; C, After cast group. All trajectories began at 0,0,0. Note the reduction in the trajectory length in B and C, and the reduction in turning in Ext B–C.

The kinematic parameters of the different classes of limb and paw movements were calculated, and comparative statistical analysis was performed only when a movement was observed at least in seven sites in two out of three groups of animals, NC movements not being considered for kinematic analysis. In ABD and EXT movement in casted rats, there was a reduction in the mean value of maximal displacement on the X, Y, and Z axes. This effect significantly involved the axis used to label the class of movement, i.e., the Y and X axes in ABD and EXT, respectively (Figure 5), whereas the effect did not emerge in ADD (Figure 5). Likewise, in OPN there was a reduction in the mean value of maximal displacement on the X and Z axes (Figure 5), whereas the two samples of OCS expressed a trend conforming to the OPN movement (Figure 5). Examination of other kinematic parameters revealed that long-term cast-immobilization had no major effects on the latency or duration of any class of movement (Tables 3, 4). Conversely, the long-term cast produced a strong reduction in the maximal speed, in the average speed, and also trajectory length (Figure 6) and displacement vector length. In limb movements (Table 3), the effect was marked in ABD and EXT, but less pronounced on ADD values. In paw movements (Table 4), the effect was marked in OPN maximal velocity, but did not influence average speed. The two samples of OCS expressed a trend compliant with the other movements. As regards trajectories, in controls all were curved (path index, >1) and in 72.46% the curvature was greater than that of a circle (path index, >1.57). The trajectories of animals subjected to casting were also curved in all cases, but the percentage of trajectories whose curvature exceeded that of a circle decreased to 45.07%. Thus, the reduction in length was accompanied by a reduction in the curvature or turning of trajectories (example: Figure 6, EXT).

**Table 3:** **Mean scores (±s.e.m.) for kinematic variables of classes of limb movement in Control, Cast, and After cast groups**.

**Class**	**L(ms)**	**D(ms)**	**MPV(mm/s)**	**MV(mm/s)**	**T(mm)**	**DV(mm)**
**CONTROL GROUP**						
ABD_*n* = 83_	23.47 ± 0.97	499.60 ± 13.64	372.21 ± 20.33	107.95 ± 6.59	57.73 ± 3.71	26.24 ± 1.14
ADD_*n* = 15_	24.90 ± 1.87	470.10 ± 37.10	386.41 ± 49.77	91.68 ± 11.51	39.23 ± 3.26	13.76 ± 1.65
EXT_*n* = 37_	21.30 ± 1.10	444.77 ± 14.92	381.77 ± 31.31	145.77 ± 14.13	68.63 ± 8.20	21.40 ± 1.62
**CAST GROUP**						
ABD_*n* = 50_	21.88 ± 1.55	457.73 ± 12.04	151.13 ± 10.71^***^	50.50 ± 4.53^***^	22.49 ± 1.80^***^	12.39 ± 0.60^***^
ADD_*n* = 4_	20.00 ± 0.00	514.58 ± 23.07	92.00 ± 22.05^**^	35.14 ± 6.55	18.80 ± 2.38	14.91 ± 2.69
EXT_*n* = 11_	20.00 ± 0.00	426.97 ± 30.50	200.96 ± 33.69^**^	71.51 ± 10.46^*^	31.43 ± 5.03^*^	11.43 ± 1.15^***^
**AFTER CAST GROUP**						
ABD_*n* = 54_	21.31 ± 0.59	456.20 ± 11.86	221.89 ± 14.57^***^^◦^	72.49 ± 5.56^***^	30.32 ± 2.08^***^	16.34 ± 0.75^***^^◦^
ADD_*n* = 12_	21.67 ± 1.67	505.97 ± 31.06	208.68 ± 32.17^*^	79.75 ± 13.64	41.45 ± 7.82	16.39 ± 1.54
EXT_*n* = 36_	20.00 ± 0.00	410.00 ± 13.52	269.15 ± 24.60^*^	135.38 ± 16.39	52.44 ± 6.09	15.09 ± 0.88^**^

ABD, abduction; ADD, adduction; and EXT, extension. Kinematic variables: L, movement latency; D, movement duration; MPV, maximum peak velocity; MV, mean velocity; T, trajectory; DV, displacement vector. Units: ms, milliseconds; s, second; mm: millimeters; n, number of sites for each class. The kinematic parameters were calculated, and comparative statistical analysis was performed only when a class was observed at least seven sites in two out of three groups of animals. Statistical comparison:

* P < 0.05,

** P < 0.01,

*** P < 0.001, different from control;

◦ P < 0.05, After cast different from Cast group (ANOVA followed by contrast analysis and the sequentially rejective Bonferroni test for multiple comparisons).

**Table 4:** **Mean scores (±s.e.m.) for kinematic variables of the classes of paw movement in Control, Cast, and After cast groups**.

**Class**	**L(ms)**	**D(ms)**	**MPV(mm/s)**	**MV(mm/s)**
**CONTROL GROUP**				
OPN_*n* = 60_	24.50 ± 1.35	399.33 ± 14.35	271.49 ± 22.55	33.25 ± 2.67
OCS_*n* = 10_	25.00 ± 1.67	546.00 ± 41.72	352.99 ± 43.10	88.32 ± 15.91
**CAST GROUP**				
OPN_*n* = 18_	24.44 ± 1.71	397.04 ± 30.27	166.80 ± 28.20*	31.33 ± 5.05
OCS_*n* = 2_	20.00 ± 0.00	442.50 ± 7.50	229.29 ± 4.05	78.44 ± 19.73
**AFTER CAST GROUP**				
OPN_*n* = 33_	21.40 ± 0.77	412.32 ± 16.67	170.10 ± 9.64**	30.84 ± 2.87
OCS_*n* = 13_	21.54 ± 1.54	435.64 ± 21.90	290.88 ± 34.81	108.22 ± 13.55

Classes of paw movement: OPN, opening; OCS, opening/closure sequence. See Table 3 for kinematics.

### Long-term cast altered ICMS-defined reachable space

**Figure 7:**
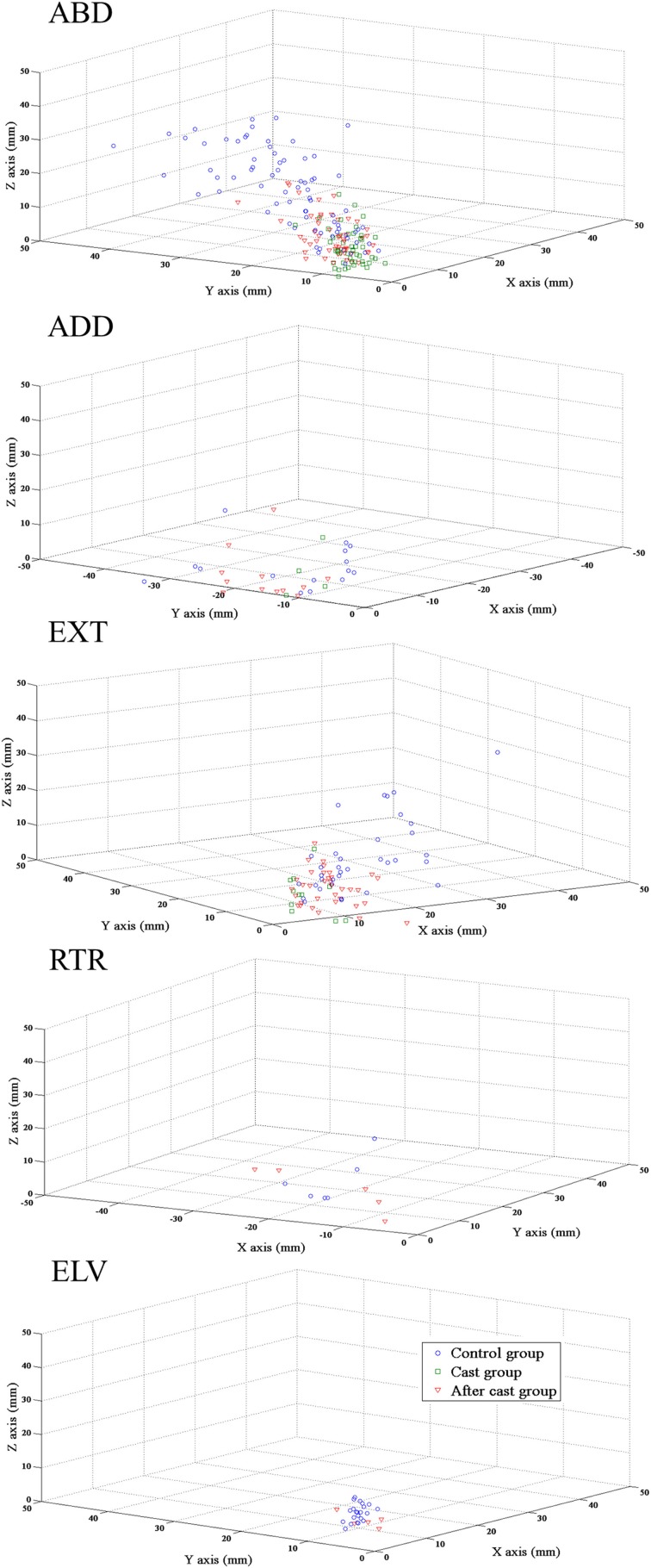
**Unilateral forelimb casting affected the spatial endpoint dispersion for limb movement classes**. Scatter plots showing endpoint dispersion of limb movements in 3D space for all animals. Individual endpoints are represented by a symbol. The rat groups are color-coded as follows: blue, Control group; green, Cast group; red, After cast group. ABD: abduction, ADD: adduction, EXT: extension, RTR: retraction, ELV: elevation. All movements began at 0,0,0. Note that scales and calibrations are not equivalent on each axis; this improves the legibility of the graph but reduces the apparent amplitude of the Z axis. Final endpoint locations were different for each class of movement, and cast-immobilization caused endpoint clustering within a narrow space.

**Figure 8:**
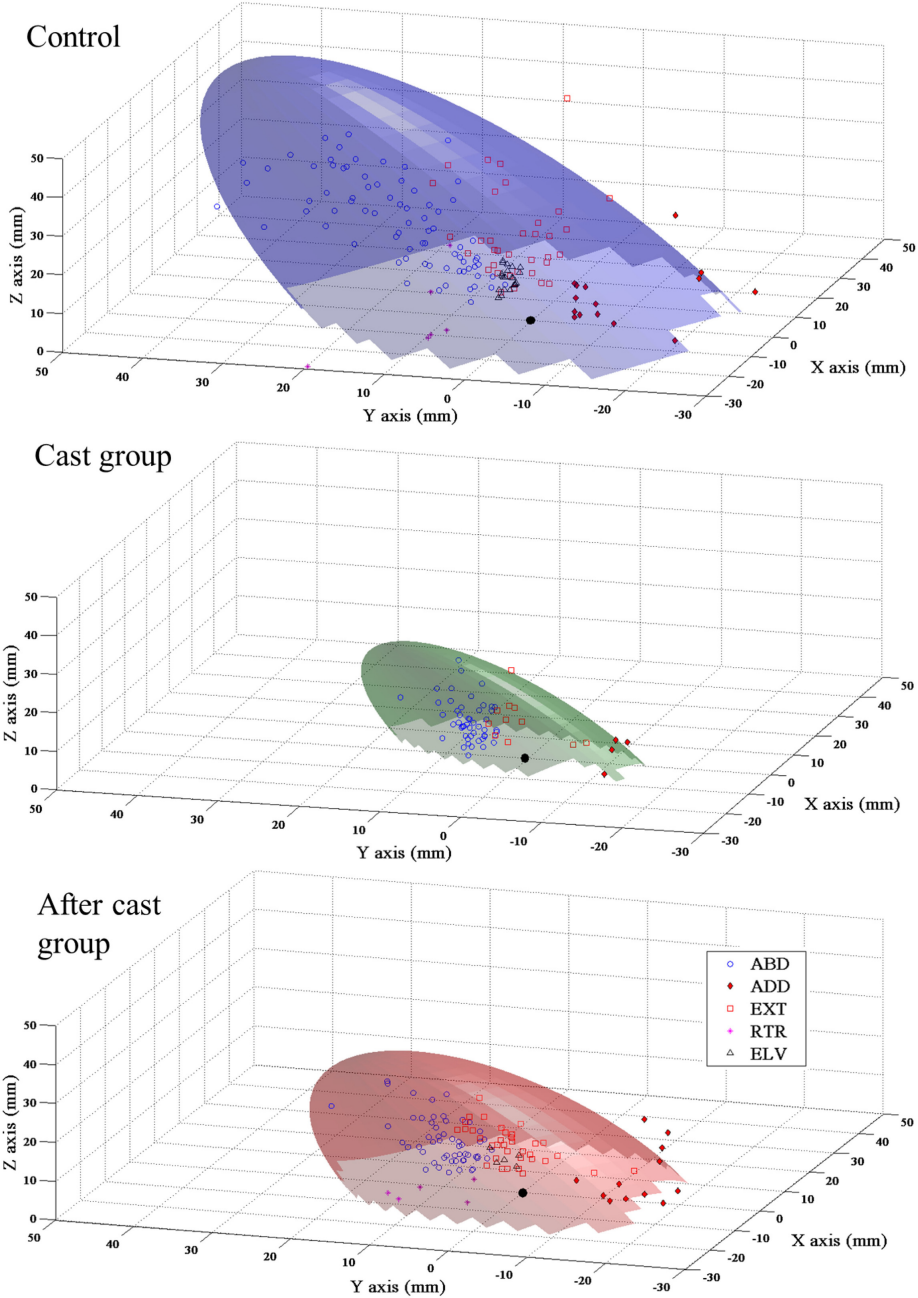
**Unilateral forelimb casting affected the representation of ICMS-defined reachable space**. Plastic representation of the ICMS-evoked reachable space in Control, Cast and After cast groups. Abbreviations: as in the Figure 7. Each plot represents the volume that included 95% of all endpoints of movements evoked in the group of animals. Symbols: endpoints of movements; filled black circle: starting point at 0,0,0 of coordinate. NC movement was not considered in this analysis. Note that scales and calibrations are not equivalent on each axis; this improves the legibility of the graph but decreases the apparent amplitude of the Z axis.

In rats, the most consistent feature of ICMS-evoked movements was limb displacement toward a region of reachable space (Bonazzi et al., 2013). To assess how limb-evoked movements defined features of reachable space, the endpoints of evoked movement were plotted in 3D space for each movement class. These distributions showed a clear arrangement of endpoints in different parts of space in each movement class, so that each class of reaching movement mapped a specific sector of reachable space (Figure 7). Moreover, these distributions clearly showed that long-term cast-immobilization reduced the endpoint displacement of reaching movements and caused a remapping of ICMS-reachable space features within a narrow space (Figure 7). To quantitatively characterize the features of reachable space, we plotted the endpoints of all movements obtained in all animals, and in this cumulative 3D plot we defined the volume that included 95% of the endpoint and its surface. This showed that long-term cast-immobilization was responsible for a considerable shrinkage of the surface area and volume of reachable space, since they corresponded to 21.50 and 10.04%, respectively, of control values (Figure 8, Cast vs. Control group, surface area: 1823.20 vs. 8477.90 mm^2^, volume 34,033.28 vs. 3,38,822.06 mm^3^).

### After-cast recovery of complex movement representation and kinematics

In this section, the main effects of 15 days of forelimb freedom after long-term cast-immobilization are summarized.

#### Number of sites

Number of sites at which complex forelimb movement was evoked was not different to normal (total sites *n* = 124, mean in each animal: 20.67 ± 0.49, *p* > 0.05; Figure 1). It is likely that the number of sites at which limb (*n* = 70, mean in each animal: 11.67 ± 0.80, *p* > 0.05), limb-paw (*n* = 48, mean in each animal: 8.00 ± 1.06, *p* > 0.05) and paw movement (*n* = 6, mean in each animal: 1.00 ± 0.45, *p* > 0.05) were evoked returned to baseline values during the post cast-removal period. This recovery in the number of responsive sites comprehended the entire forelimb map (Figures 2D,E).

#### Classes of movement

In line with these findings, there was a recovery in limb and paw movement classes approaching that of controls. The repertoire of evoked limb movements included: (i) ABD (*n* = 54, 45.76%); (ii) ADD (*n* = 12, 10.17%); (iii) EXT (*n* = 36, 30.51%); (iv); (v) ELV (*n* = 5, 4.24%); RTR (*n* = 5, 4.24%); and (vi) NC (*n* = 6, 5.08%). Discriminant analysis demonstrated a high rate of correct classification (predictors: X-, Y-, and Z-axis values vs. movement class; 88.98%, 105 out of 118 movements; *p* < 0.0001), and also revealed that the 11.02% false alarm rate was due to some ABD (9 out of 54) and EXT (4 out of 36) movements being recognized as ELVs. The repertoire of paw movements included: (i) OPN (*n* = 33, 61.11%); (ii) OCS, (*n* = 13, 24.07%); (iii) SUP, (*n* = 6, 11.11%); and (iv) NC (*n* = 2, 3.71%), while there was no evidence of CLO movement. Multivariate discriminant analysis demonstrated a high correct classification rate (predictors: X-, Y-, and Z-axis values vs. movement class 70.15%, 47 out of 67 movements; *p* < 0.0001). The false alarms (29.85% of movements) in this case were largely due to errors in discrimination between OPN and the OCS opening phase (classification rate: 60.61 and 53.80%, respectively).

#### Representation size

The size of cortical regions representing different classes of limb movement recovered to levels similar to control values, with the exception of ELV (Figure 3, ABD: 2.25 ± 0.24 mm^2^, *p* > 0.05, ADD: 0.50 ± 0.11 mm^2^, *p* > 0.05, EXT: 1.50 ± 0.14 mm^2^, *p* > 0.05, ELV: 0.21 ± 0.08, *p* < 0.01, RTR: 0.21 ± 0.12 mm^2^, *p* > 0.05). Likewise, OPN, OCS and SUP recouped their area in comparison to controls (Figure 3; OPN: 1.38 ± 0.33 mm^2^, *p* > 0.05, OCS: 0.54 ± 0.15 mm^2^ *p* > 0.05, SUP: 0.25 ± 0.11 mm^2^, *p* > 0.05). CLO, on the other hand, did not recover its full representation, while NC movement showed a slight and not significant increase in its cortical representation size (limb: 0.25 ± 0.09, *p* > 0.05; paw: 0.08 ± 0.05, *p* > 0.05). Notably, the cumulative maps showed that each movement class recovered its representation in its proper cortical area, retaining the baseline values of CoG, except for RTR at the medio-lateral coordinate (Figure 3: After-cast vs. Control group, mean shift in antero-posterior coordinate: 254.75 ± 65.71 μm; mean shift in medio-lateral coordinate: 221.00 ± 96.06 μm).

#### Combined limb-paw movements

In line with the recovery of movement classes, the After-cast group also showed a recovery of reach-shaping, reach-grasp and hold-like movements (Figure 4, After-cast vs. Control group; reach-shaping: 1.29 ± 0.29 mm^2^, *p* > 0.05, reach-grasp: 0.54 ± 0.15 mm^2^, *p* > 0.05, hold-like: 0.25 ± 0.11 mm^2^, *p* > 0.05), while no bring-to-body was observed in any mapped After-cast animals.

#### Kinematics

All After-cast movements showed a slight increase in maximal displacement values when compared to values in the Cast group, but were still lower than those found in the Control group (Figure 5). Likewise, other After-cast kinematic values (Tables 3, 4) were greater than values measured for the Cast group, and lower than those in the Control group. It should be noted that ANOVA revealed differences between ABD and EXT, with EXT having greater recovery values than ABD. Furthermore, as regards the curvature of trajectories, the After-cast group showed similar values to the Control group; all trajectories were curved (path index, >1), and in 61.02% the curvature was greater than that of a circle (path index, >1.57).

#### ICMS-defined reachable space

Fourteen days of forelimb freedom after cast removal Reachable space surface area and volume values were still considerably lower than those in Control group (3403.40 mm^2^, 84,563.49 mm^3^, i.e., 40.14 and 24.96% of that of controls, respectively) but evidently higher than those found in the Cast group (Figure 8).

## Discussion

This is the first study to show the deleterious impact of long-term forelimb disuse on the complex features of forelimb motor organization. Here we add new data that appears to strengthen the postulated link between sensorimotor activity, evoked complex movements, and their representation in the forelimb motor cortex. Three main findings were evidenced by the present study. First, the results have added new evidence of the patch-like functional zones in the rat M1, and disclosed the relationship between functional zones and ICMS-defined reachable space. Second, we show that the sensorimotor restriction of the forelimb affected complex movement representation and kinematics, in addition to the ICMS-evoked reachable space. Third, after-cast recovery of sensorimotor function restored the number of sites where complex movement could be evoked with only partial restoration of kinematic values and a slight increase in reachability parameters.

### Long-duration ICMS in control animals

As a whole, our results in control animals confirmed those of previous study (Bonazzi et al., 2013), and differences observed when the two studies are compared fall within the biological variability spectrum of observations drawn from groups with a relatively small number of animals. Current results provide further corroborative support for previous studies indicating a functional organization of motor cortex based on complex movement representations, rather than muscle somatotopy. Long-duration ICMS produced classes of complex movement that differed according to where motor cortex sites were stimulated. We document a specialization and spatial segregation of classes of movements within forelimb motor cortex otherwise unattainable using traditional short duration ICMS evoking only brief twitches of somatic musculature. A segregation in which different zones are devoted to different classes of movement is confirmed by our study as the major organizing principle of the forelimb motor cortex in rats. This functional parcellation of the forelimb cortex, presumably results from differences in intrinsic intracortical circuitry as well as afferent and efferent projection pathways (Haiss and Schwarz, 2005; Harrison et al., 2012). Moreover, the arrangement of functional zones has an orderly relationship to extrinsic space. To more clearly evidence this relationship, we measured the space encompassing the endpoints of ICMS-evoked reaching movements. Our analysis clearly showed that each class of forelimb movement represents a different portion of reachable space. Finally, it is worth discussing the nature of additional movement observed after stimulation at the border of the forelimb motor cortex, which is hindered by two factors. The first is that, due to current spread, excitation spread could be influential in determining the type of additional movement evoked together with forelimb movement. The second is that electricity will stimulate not only cell bodies and dendrites near the electrode, but also axons, including those of the cortico-cortical projections that link the forelimb cortex to neighboring representations (Huntley, 1997). In light of this, additional movement can be interpreted as a spread of stimulation current, or, alternatively, as complex movements in which coordinated forelimb movement is evoked in reaching-orienting (Erlich et al., 2011) or bringing-to-the-mouth movement (Bonazzi et al., 2013). Establishing criteria to correctly distinguish between these two scenarios is important for future experiments.

### Long-duration ICMS in cast animals

The reorganization of forelimb cortex disclosed by long-duration ICMS primarily involved a strong reduction in the number of sites at which complex movement could be evoked. This change reflects a strong decrease in the excitability of the cortico-spinal tract and indicates relevant alterations in sensorimotor cortical processing (Langlet et al., 2012; Viaro et al., 2014). Nonetheless, the M1 remodeling brought about by immobilization predominantly affected the representation of paw movement classes. This suggests that within the motor cortex, where distal- and proximal-projecting forelimb neurons are intermingled (Wang et al., 2011), the distal-projecting forelimb neurons lose their excitability to a greater extent than the proximal-projecting neurons. The analysis of movements sought to define the classes of movement according to the maximal displacement in one of the X-, Y- or Z-axes. We found that forelimb casting affected maximum displacement in X-, Y-, and Z-axes for limb and paw classes of movement though evoked movements in cast-immobilized rats were well classified in classes using the control criteria. ICMS studies resulted in two possible interpretations of motor cortex organization that depend upon how electrical stimulation activates the motor circuits. Short-duration ICMS provided a threshold-map of body musculature (Asanuma and Rosen, 1972; Donoghue and Wise, 1982) whereas long-duration ICMS provided complex, multi-joint, movements resembling those of natural behavior (Graziano et al., 2002a, 2005; Haiss and Schwarz, 2005; Ramanathan et al., 2006; Bonazzi et al., 2013). Convergent experimental observations have shown considerable plasticity of M1 movement representation as defined by short-duration ICMS (Kaas, 2000; Sanes and Donoghue, 2000). Motor mapping has revealed changes resulting from motor nerve injury (Sanes et al., 1990; Toldi et al., 1996; Franchi, 2000) loss of muscle activity (Sanes et al., 1990; Cohen et al., 1991; Franchi, 2002) and skill learning (Nudo et al., 1996; Kleim et al., 1998; Plautz et al., 2000; Coq et al., 2009). While skill learning caused an increase in area of the trained forelimb and a decrease in area of the under-trained forelimb, total forelimb area remaining constant, nerve injury and muscle inactivity both resulted in expansion of the neighboring intact representation into the disconnected cortical representation. Conversely, the whole limb sensorimotor restriction profoundly increased thresholds and shrunk the limb representation area, without compensation by the neighboring cortex, leaving a part of the limb representation unresponsive to short-duration electrical stimulation (Langlet et al., 2012; Viaro et al., 2014). The present study clearly shows that whole limb sensorimotor restriction profoundly altered kinematics and shrunk the forelimb representation of complex movement, leaving part of the forelimb representation unresponsive to long-duration ICMS. Thus, we can conclude that a long period of limb disuse altered output to muscles as well as complex features of M1 organization. Previous long-duration ICMS studies suggest that all complex forelimb movements found in reach-trained rats were observed in untrained controls (Ramanathan et al., 2006; Brown and Teskey, 2014). Similarly, reach training did not alter either the size or location of complex forelimb movement representation. However, it can't be ruled out that skilled training could change kinematic variables of evoked complex movements without relevant changes in the type and topography of movement.

Parcellation of the motor cortex into functionally distinct zones has been described for the forelimb in monkeys (Graziano et al., 2005; Gharbawie et al., 2011), rats (Bonazzi et al., 2013), and mice (Harrison et al., 2012). In our study, one of the most striking findings was that the sensorimotor restriction reduced the size of the cortical zones, while recovery of sensorimotor function brought about a restoration of their size in the proper cortical territory.

Up today there is uncertainty as to what the complex movements produced by long-duration stimulation actually means behaviorally. Recent papers suggested that the long-duration stimulation hijacks the motor cortical circuitry, replacing natural activity with activity that is largely stimulus driven (Griffin et al., 2011, 2014). In the current study, there was concurrent production of distal with the more proximal components of the movement that reflected proximal/distal coactivation rather than fragments of actual behaviors. Conversely, the most consistent feature of limb and paw movements was their combination in specific patterns which may have a behavioral relevance (Graziano et al., 2002a, 2005; Bonazzi et al., 2013). Moreover, these findings highlighted different functional regions characterized by different sensitivity for movement restriction, with loss of representation being greater in zones of the cortex representing manipulative activity within the immediate body sphere. For example, the forelimb map clearly showed a large reduction in the representation of reach-shaping and reach-to-grasp/hold-like movements, normally represented in the rostral and lateral parts of the forelimb cortex. The preserved partial limb movement in the caudal part of the forelimb motor cortex (ABD and EXT) could be consistent with the rapid post-cast recovery of the forelimb use in postural support and walking (Viaro et al., 2014). Sensorimotor restriction induced changes in kinematics that were observed in movements elicited from all cortical sites. These changes were reversible, although recovery of the control group values remained partial 2 weeks after cast removal. In fact, all movements showed decreases in maximal velocity, trajectory, and vector length, but no change in latency or duration. It should be noted that these changes could be due to cortical or spinal differences in how the movements were induced by long-duration ICMS. They were most likely due to cortical effects, since changes in spinal cord circuitry excitability are related to changes in the latency and duration of cortical ICMS-evoked movements (Ahmed, 2013), which remained unchanged in this experiment. Thus, decreasing movement velocity and amplitude indicate that sensorimotor restriction reconfigures motor circuitries to recruit, more slowly, fewer cortico-spinal neurons over time. This also relates to the finding that trajectories of the same duration were shorter, straighter, and with less pronounced turning in Cast and After-cast animals in comparison to Controls. Conversely, latency and duration could be due to the direct activation of corticospinal neurons, being, therefore, directly dependent on the parameters of the stimulus (Histed et al., 2009; Logothetis et al., 2010; Griffin et al., 2011, 2014).

Yet it is possible that ICMS as used in the present experiments activates directly the most excitable elements of the motor cortex and that these elements tend to project to other cortical areas as well as subcortical regions (cerebellum and basal ganglia), connected monosynaptically to the directly excited cortical neurons (Logothetis et al., 2010) and involved in the generation of the forelimb movements. Specifically, present findings may call into question modifications taking place in the cerebellum, which have shown to have short-latency effects on motor cortex excitability (Sultan et al., 2012). Moreover, it is known that prolonged cast immobilization reduces the forelimb somatosensory cortex (S1) excitability in parallel with the corresponding representation in motor regions (Coq and Xerri, 1999; Lissek et al., 2009). Thus, a loss of excitability of the forelimb S1 and its connection with the forelimb motor cortex (Kim and Lee, 2013) could also explain the alterations in complex cortically elicited movement. In addition, as we did not assess the effects at spinal and at muscular level, we cannot exclude other subcortical contributions along the motor system. Indeed, muscles could be weakened and joints could be altered after weeks of cast immobilization.

In order to discuss plasticity effects in space representation after long-term cast immobilization, we refer to well-documented sensory-to-motor functions of body and peripersonal space representation. It is well known that information related to the position of different body parts and stimuli within peripersonal space are directly linked to the motor system in both monkeys (Rizzolatti et al., 1997; Graziano and Cooke, 2006) and humans (Coello et al., 2008; Serino et al., 2009; Canzoneri et al., 2013). Consistently, we show here in rats that limiting the possibility of forelimb action led to a contraction of ICMS-induced reachable space representation, which was reversed after restoring the potential for such movement. However, since there is uncertainty as to what the representations produced by long-duration stimulation means behaviorally, the extent to which altered reachable space processing can be ascribed to changes in the body schema (Cardinali et al., 2009), or perceived position of the limb (Brozzoli et al., 2012), still remain to be determined.

Although these experiments did not directly test specific motor circuits, the theoretical implication of the current study is that sensorimotor experience has a significant role in shaping complex cortically elicited movement. The results suggest that relevant features of complex movement such as distal-proximal combination and spatial coordination are controlled by circuits sensitive to experience. That is to say, relevant and specific aspects of complex movement features may be learned and stored within parallel re-entrant systems to motor cortex. Future studies are needed to reveal the influence of specific motor circuits on ICMS-evoked behaviorally relevant movements.

### Conflict of interest statement

The authors declare that the research was conducted in the absence of any commercial or financial relationships that could be construed as a potential conflict of interest.
